# Pesticide Residues in Peri-Urban Bovine Milk from India and Risk Assessment: A Multicenter Study

**DOI:** 10.1038/s41598-020-65030-z

**Published:** 2020-05-15

**Authors:** J. P. S. Gill, J. S. Bedi, Randhir Singh, Mohd Nadeem Fairoze, R. A. Hazarika, Abhishek Gaurav, Sudhir Kumar Satpathy, Abhimanyu Singh Chauhan, Johanna Lindahl, Delia Grace, Amit Kumar, Manish Kakkar

**Affiliations:** 1 School of Public Health and Zoonoses, Guru Angad Dev Veterinary and Animal Sciences University, Ludhiana, 141004 Punjab India; 2 Karnataka Veterinary, Animal and Fisheries Sciences University, Nandinagar, Bidar, 585 401 Karnataka India; 3 Assam Agricultural University, Khanapara, Guwahati, 781 022 Assam India; 4 Rajasthan University of Veterinary and Animal Sciences, Veterinary University Road, Near Deen Dayal Upadhyay Circle, Bikaner, 334001 Rajasthan India; 5 KIIT School of Public Health, Bhubaneswar, 751024 Odisha State India; 6 Public Health Foundation of India, Plot 47, Sector 44, Gurugram, Haryana India; 7 International Livestock Research Institute, Box 30709, Nairobi, Kenya; 8 Zoonosis Science Centre, Uppsala University, Uppsala, 751 23 Sweden; 9 Swedish University of Agricultural Sciences, Uppsala, 750 07 Sweden

**Keywords:** Chemical biology, Environmental sciences

## Abstract

Pesticides residue poses serious concerns to human health. The present study was carried out to determine the pesticide residues of peri-urban bovine milk (n = 1183) from five different sites (Bangalore, Bhubaneswar, Guwahati, Ludhiana and Udaipur) in India and dietary exposure risk assessment to adults and children. Pesticide residues were estimated using gas chromatography with flame thermionic and electron capture detectors followed by confirmation on gas chromatography-mass spectrometer. The results noticed the contamination of milk with hexachlorocyclohexane (HCH), dichloro-diphenyl trichloroethane (DDT), endosulfan, cypermethrin, cyhalothrin, permethrin, chlorpyrifos, ethion and profenophos pesticides. The residue levels in some of the milk samples were observed to be higher than the respective maximum residue limits (MRLs) for pesticide. Milk samples contamination was found highest in Bhubaneswar (11.2%) followed by Bangalore (9.3%), Ludhiana (6.9%), Udaipur (6.4%) and Guwahati (6.3%). The dietary risk assessment of pesticides under two scenarios i.e. lower-bound scenario (LB) and upper-bound (UB) revealed that daily intake of pesticides was substantially below the prescribed acceptable daily intake except for fipronil in children at UB. The non-cancer risk by estimation of hazard index (HI) was found to be below the target value of one in adults at all five sites in India. However, for children at the UB level, the HI for lindane, DDT and ethion exceeded the value of one in Ludhiana and Udaipur. Cancer risk for adults was found to be in the recommended range of United States environment protection agency (USEPA), while it exceeded the USEPA values for children.

## Introduction

The usage of pesticides has not only provided an important benefit to agricultural production and crop protection but at the same time played a significant role in prevention and control of vector-borne human and animal diseases. However, the occurrence of pesticide residues in several components of the environment and food commodities has raised grim concerns about their use^1,2^. The presence of pesticides remains in food articles is considered a worldwide public health concern and one of the foremost causes of issues related to international trade^3^. Despite a low per hectare use of pesticides in India, their injudicious use has led to the presence of residues in both biotic and abiotic sections of the environment^4^. Until the restrictions on the use of Organochlorine pesticides (OCPs) in late 1990s, dichloro-diphenyl trichloroethane (DDT) and hexachlorocyclohexane (HCH), were highly used pesticides in India^5^. Still, India has been permitted to use a substantial amount of DDT for the control of vector borne disease such as malaria^6^. In India, with the changing agricultural practices, consumption of pesticides belonging to organophosphate (OPs) and synthetic pyrethroid (SPs) groups has increased many folds^7,8^, which is evident by their presence in in vegetables, animal feed and even in human breast milk^9–12^.

Being a good source of proteins, fat and minerals bovine milk is an essential component in the diet of humans of all ages. India’s dairy production is about 164 million tonnes in 2016-17 and milk is India’s largest crop worth around USD 90 billion which is much more than rice and wheat put together, with higher export potential. Milk producing animals are exposed to pesticides either through dietary intake of feed and fodder possessing their residues or by application of pesticides for ectoparasite control on the animal body, animal sheds and milk processing areas^13^. Because of the persistence and lipophilic nature, pesticides results in magnification of their residues in fat-rich tissues of animals. During lactation the mobilization of deposited contaminants in animal fats resulted in their excretion in milk^14^.

An increasing number of pesticides are contaminating the environment day by day considered to be potentially unsafe to human/animal health as well as to the ecosystem^15^. Long-term exposure to pesticides may cause kidney and liver problems^16^, disruption of the endocrine system^17^, neurological and immune system disorders^18^ and higher risk of lungs, breast, cervix and prostate cancer^19^. Further, pesticides contamination to the local environment may also affect birds, wildlife, aquatic and domestic animals population. It is hypothesized that toxic effects of acute exposure to pesticides in animals and humans are easily recognized, but the long term exposure to low doses through dietary intake are not easy to distinguish. The effects of a regular intake of pesticide residues through food are difficult to identify and quantify. Thus exposure as well as risk assessments are necessary to determine the effects originated because of continuous intake of pesticides residue in food. Therefore, it is necessary to ascertain the levels of contamination of food matrices, so that human exposures to these contaminants, particularly by dietary intake, do not exceed the recommended acceptable limits for health.

The presence of OCPs residues in food of animal origin comprising meat, milk and their products has been widely documented in India^20–22^ but scattered studies have reported multiple pesticide residue contamination comprising of OCPs, OPs and SPs^23,24^. There’s a dearth of comprehensive studies that are representative of different parts of India in terms of residues trend as well as health hazards linked with consumption of milk contaminated with pesticides residue. Population and economic growth have fostered urbanization in the country with rising food demands. The dairy farming is centralized in the peri-urban fringes to supply milk and milk products to the urban consumers. The urban population in India has increased from 25.7% in year 1991 to 31.16% in year 2011^25^. To meet the growing demands of food for human and animal feed for peri-urban lactating animals, the agricultural practices are turning towards modern and industrialized farming^26,27^. Moreover, to minimize the incidence of vector borne diseases and for control of ectoparasite in peri-urban intensive farming system, insecticides are often used.

Thus, keeping in mind the improper use of pesticides, their changing consumption patterns, the restrictions on the use of persistent OCPs (HCH, DDT, endosulfan), and the lack of information and awareness on contamination of bovine milk with OP and SP pesticides in urban vicinities, the present study aimed to quantify OCPs, SPs, and OPs in milk produced in the peri-urban fringes from five different cities in different parts of India namely, Bangalore, Bhubaneswar, Guwahati, Ludhiana and Udaipur to get better geographical representation.

## Results and discussion

Since, milk and milk products are widely consumed by the infants, children, and adults throughout the world therefore the contamination of milk has become a matter of serious concern.

### Contamination patterns of pesticide residues in peri-urban bovine milk

The pesticide residue analysis of milk from all five sites indicated the occurrence of organochlorines (β-HCH, lindane, endosulfan and DDT), organophosphates (ethion, profenofos, chlorpyrifos), synthetic pyrethroids (cyhalothrin, cypermethrin, permethrin) and phenylpyrazole (fipronil) residues. Among all the 1183 milk samples, 93 (7.9%) were noticed with detected residues (>limit of detection) and 1090 (92.1%) were noticed to be undetected/not detected (<limit of detection). The number of peri-urban farm’s milk positivity for any type of pesticide residues reflected highest contamination in Bhubaneswar (11.2%) followed by Bangalore (9.3%), Ludhiana (6.9%), Udaipur (6.4%) and Guwahati (6.3%) (Table 1). Between organochlorines, DDT (p,p′ DDE, p,p′ DDD and o,p′ DDE) was predominant in peri-urban milk at all five sites. However, chlorpyrifos and cypermethrin were the most common organophosphates and synthetic pyrethroids, respectively (Table 2). Since the milk samples were collected from individual animals in a farm, the farm level prevalence was calculated based on the milk sample positivity even of one animal among selected. Thus, in Bangalore, Bhubaneswar, Guwahati, Ludhiana and Udaipur, out of 102 farms at each site, 19.6, 22.5, 16.6, 17.6 and 14.7 per cent farms were positive for pesticide residues, respectively.

**Table 1:** Prevalence of pesticide residues in peri-urban milk at five sites in India.

Study Site	Number of milk samples tested	Number of milk samples positive	Percentage positive
Bangalore	216	20	9.3
Bhubaneswar	204	23	11.2
Ludhiana	258	18	6.9
Guwahati	270	17	6.3
Udaipur	235	15	6.4
Total	**1183**	**93**	**7.9**

**Table 2:** Distribution of pesticide residue levels (ng/g ) in milk from five different sites of India.

Pesticide	Bangalore (n = 216)		Bhubaneswar (n = 204)		Ludhiana (n = 258)		Guwahati (n = 270)		Udaipur (n = 235)	
	Mean ± SD (Max)	Positive	Mean ± SD (Max)	Positive	Mean ± SD (Max)	Positive	Mean ± SD (Max)	Positive	Mean ± SD (Max)	Positive
β-HCH	0.35 ± 3.02 (31)	3	ND	—	0.22 ± 2.39 (35.0)	3	0.45 ± 5.2 (67.0)	2	ND	—
Lindane	0.76 ± 5.1 (42.0)	5	ND	—	0.09 ± 1.5 (18.0)	2	0.79 ± 7.3 (85.0)	4	0.17 ± 2.6 (40.0)	1
p,p′DDE	0.53 ± 5.3 (75.0)	4	0.30 ± 2.2 (20.0)	4	0.19 ± 2.19 (29.0)	2	1.53 ± 10.5 (108.0)	7	0.43 ± 4.0 (50.0)	3
o,p′ DDD	ND	—	1.2 ± 12.9 (170.0)	3	0.05 ± 0.8 (13.4)	1	0.19 ± 2.2 (30.0)	2	0.22 ± 2.4 (27.0)	2
Σ DDT	0.53 ± 5.3 (75.0)	4	1.60 ± 13.1 (170.0)	7	0.24 ± 2.34 (29.0)	3	1.70 ± 10.7 (138.0)	9	0.60 ± 4.6 (77.0)	5
Endosulfan	1.24 ± 9.2 (86.0)	4	0.20 ± 2.0 (22.0)	2	ND	—	1.21 ± 10.7 (130.0)	4	ND	—
Chlorpyrifos	1.71 ± 11.1 (81.0)	5	1.30 ± 9.1 (71.0)	4	1.57 ± 10.3 (85.0)	6	0.76 ± 6.5 (65.0)	4	1.62 ± 12.7 (130.0)	4
Ethion	1.05 ± 8.9 (86.0)	3	0.68 ± 6.7 (73.0)	2	0.80 ± 7.4 (74.0)	3	1.46 ± 20.0 (320.0)	2	1.02 ± 9.0 (83.0)	3
Profenophos	0.94 ± 8.0 (74.0)	3	0.32 ± 4.6 (66.0)	1	0.78 ± 7.2 (71.0)	3	0.25 ± 4.1 (68.0)	1	0.31 ± 4.8 (73.0)	1
Cypermethrin	1.20 ± 8.3 (76.0)	5	ND	—	1.74 ± 21.4 (340.0)	5	0.39 ± 3.7 (44.0)	3	ND	—
Permethrin	0.31 ± 3.4 (45.0)	2	11.1 ± 74.2 (650.0)	5	0.30 ± 2.8 (32.0)	3	1.80 ± 22.9 (370.0)	4	28.17 ± 172.3 (1840.0)	10
Cyhalothrin	0.11 ± 1.18 (15.0)	2	0.39 ± 4.07 (50.0)	2	0.62 ± 6.9 (102.0)	3	0.79 ± 8.3 (120.0)	3	0.17 ± 2.6 (40.0)	1
Fipronil	1.17 ± 7.6 (75.0)	6	ND	—	0.76 ± 7.7 (110.0)	4	0.30 ± 3.5 (50.0)	2	0.34 ± 5.2 (80.0)	1
Butachlor	ND	—	15.1 ± 154.08 (1760.0)	2	ND	—	ND	—	ND	—
Methoxychlor	ND	—	ND	—	ND	—	0.24 ± 2.9 (40.0)	2	ND	—
Endrin	ND	—	ND	—	0.24 ± 3.20 (50)	2	ND	—	0.09 ± 1.3 (20.0)	1

ND= Not Detected.

The contamination of bovine milk observed in the present study is lower than the earlier reports from India, that have reported comparatively higher number of contaminated milk samples as well as higher concentrations of contaminants predominately DDT and HCH^22,28,29^. As reported by Sharma *et al*., (2007) contamination of milk samples was DDT (100%), HCH (97%), endosulfan (43%), and aldrin (12%)^30^. Although DDT use has been banned in India, still the restricted usage of DDT (up to 10,000 tons/annum) to control vector-borne diseases is permitted especially in and around urbanized areas^31^. Among various DDT metabolites, p,p′ DDE was the leading owing to its lipophilicity and thus long- term bioaccumulation in living beings, which is also evident from one of our earlier studies carried out in Punjab; where p,p′ DDE, p,p′ DDT and p,p′ DDD residues were reported in human breast milk samples^10^. Nevertheless, technical HCH (mixture of α-, β-,γ- and δ- isomers) has been banned in India since 1997, still peri-urban milk samples exceeded the MRLs for γ-HCH which could be corroborated to the exemption of γ-HCH isomer use in agriculture crops including animal fodder till the imposition of ban in 2015^32^. The presence of endosulfan in milk samples could be related to the occurrence of endosulfan residues in feed and fodder meant for animals which has been reported earlier by many workers^33,34^. We were not able to detect residues of other OCP such as aldrin, chlordane, dieldrin, heptachlor, mirex and methoxychlor in any milk sample which is likely due to the imposition of ban by government of India on their use in crop protection for the last three decades.

Chlorpyriofs, ethion and profenophos were the OPs observed in peri-urban milk samples. In recent past, many researchers have noticed the presence of OP residues in milk^35,36^ related to their ability to covalently link with the milk proteins^37^. In Punjab, 6.71% milk samples were seen to be contaminated with residues of chlorpyrifos at above MRLs values^38^, similarly, Kathpal *et al*., (2001) informed the occurrence of chlorpyrifos in 03 milk samples which exceeded the MRLs^39^_._ The widespread contamination of market vegetables with OPs was seen in Hyderabad, where chlorpyrifos, triazophos, acephate, fenitrothion and phosalone were detected in eggplant, cabbage, cauliflower, tomato and ladyfinger^9^.

Synthetic pyrethroids are categorized as hydrophobic compounds having log octanol–water partition coefficient near to 06 along with less environmental persistence ranging from 12 to 197 days^40^. In present study, presence of SP residues (cypermethrin, cyhalothrin ‘and permethrin) was seen in milk samples. In India, pyrethroids are among widely used pesticides to control pests in agriculture crops, animal farms, homes, residential communities, restaurants and hospitals. Residues of cypermethrin, deltamethrin, and fenvalerate have been reported in various food products available in India^41–43^ and even in human breast milk samples in recent studies^10,44^. The, occurrence of pyrethroids in milk samples thus may be associated with their increasing use as a replacement for organochlorines^6^.

The insecticide fipronil is commonly used in control of many soil and foliar insects on a variety of crops along with flea and tick spray formulation for domestic animals. Fipronil can be excreted in milk if lactating animals are fed with feed or fodder contaminated with it^45^. The overall residue pattern observed in present study reported the predominance of OPs and SPs pesticides in milk at all sites. Thus, the present results may indicate the changing trends in use of OPs and SPs as an alternate for OCPs which is also evident in some recent studies conducted in India.

### Spatial variation for pesticide residues at different sites

The comparative pesticide residue mean levels in peri-urban bovine milk at five different sites reflected that HCH, DDT and ethion were dominant in Guwahati, while cyhalothrin, chlorpyrifos and profenophos were predominant in Bangalore and cypermethrin in Ludhiana (Table 2). Permethrin residues were exclusively detected highest in Bhubaneswar and Udaipur bovine milk samples. In Bangalore, out of 216 milk samples, fipronil residues were detected in 06 samples; chlorpyrifos, cypermethrin and lindane were observed in 05 samples each. Residues of p,p′ DDE were recoded highest in 04 samples which were also found to possess endosulfan. In regard to contamination levels, chlorpyrifos dominanted with mean levels of 1.71 ng/g followed by endosulfan (1.24 ng/g) and cypermethrin (1.20 ng/g). In Bhubaneswar, among 205 milk samples, two samples were observed to be extremely contaminated with butachlor residues (1330, 1760 ng/g) and permethrin was detected in 5 samples with aggregate mean level of 11.1 ng/g. In addition, residues of endosulfan, p,p′ DDE, o,p′ DDD, cyhalothrin, chlorpyrifos, ethion and profenophos were also observed in traces. Despite the limited use of pesticide, i.e 183 tonnes (0.06 kg/hectare) in Guwahati per year^46^, DDT residues (p,p′ DDE and o,p′ DDE) were seen in 9 out of 270 samples. Some of the samples were also found positive for HCH (lindane and β-HCH), endosulfan, methoxychlor, permethrin, cypermethrin, cyalothrin, fipronil, chlorpyrifos, ethion and profenofos. In Ludhiana site, out of 258 milk samples, residues of cypermethrin and chlorpyrifos were detected in 06 samples each followed by p,p′ DDE (05), fipronil (04). In Punjab, the total pesticide usage is highest among the five selected study sites, i.e 5743 tones (0.74 kg/hectare), almost 10% of the total pesticide usage of India^47^. In terms of concentrations of different contaminants cypermethrin and chlorpyrifos were found out to be the major ones followed by ethion, profenofos, fipronil and cyhalothrin. In Udaipur, on the contrary, comparative levels of pesticide residues were found less except of the permethrin residue, which was observed in 10 milk samples. However, the total pesticide consumption of the state of Rajasthan is 2475 (0.05 kg/hectare). Beside, permethrin, chlorpyrifos, DDT, HCH and ethion were some other pesticide that were detected in present study and also reported in previous studies of the region.

The pesticide residues distribution did reflect the similar pattern of milk contamination with limited variation. The scientific reports indicated presence of pesticide residues in dairy as well as other food products. In Bangalore, chlorpyrifos has been predominantly detected in locally sold vegetables^48^. In an Indian Agricultural Research Institute (ICAR) report published in 2014–15, about 25% samples of different food commodities from Bangalore were found positive for pesticides^49^. Similarly, human blood samples were found to be positive for HCH isomers and DDT metabolites where lindane (γ-HCH) and p,p′-DDE were the most contributor to the total OCP^50^. In Bhubaneswar residues of butachlor, the herbicide whose consumption has been increased from 380 metric tonnes to 1291 metric tonnes in a period from 2005–2010^8^, have been detected in rice grains and rice straws, which are used as animal feed that could lead to their excretion in animal milk^51^. Further, residues of endosulfan, cypermethrin and propfenofos have also been noticed in honey bees and soil samples from this region of Odisha state^52^. DDT, aldrin, endosulfan. butachlor, permethrin, cypermethrin and HCH have been reported in fish samples from Guwahati and human breast milk in North-East, India^53,54^. Organochlorine pesticides (DDT and HCH) are still being used not only in malaria control program but in agricultural fields as well; which is suggested by their presence in human breast milk samples collected from two districts of Assam i.e. Dibrugarh and Nagaon at higher levels^55^. Residues of p,p′ DDE, p,p′ DDT and p,p′ DDD have also been seen in human breast milk samples from Ludhiana, Punjab^10^. In few other findings Cheema *et al*., (2004), reported the milk samples contaminated with chlorpyrifos^38^, while Bedi *et al*., (2015) observed that few milk samples exceeded the maximum residue limits (MRLs) for γ- HCH, DDT and chlorpyrifos^23^. In Rajsthan state of India John *et al*.^56^, reported contamination of bovine milk in Jaipur city with DDT and HCH residues^56^. In a recent study by Sharma *et al*., (2015) some lakes of Rajasthan were also found to be contaminated with DDT, HCH and aldrin residues^2^.

### Risk assessment by dietary intake of contaminated milk

The assessment of pesticide residues dietary exposure depends upon the presence of its residue in food and availability/consumption rate of that food which is compared with the health-based toxic reference values such as acceptable daily intake values (ADI) and reference doses (RfD) for both short as well as long- term risk assessment, respectively^57^. The estimated average daily dietary intake values of detected pesticides in this study were observed below the recommended ADI values in all five sites at both lower bound and upper bound scenarios except for fipronil in children belonging to Ludhiana and Udaipur at upper bound (Table 3). Similarly, comparative higher EDI values were observed for residents of both Ludhiana and Udaipur could be due to higher availability of milk and milk products in these two regions as compared to other three. In Bangalore, the higher EDI was seen for fipronil (85.5 and 14.2% of ADI in children and adult, respectively) followed by ethion (13.5; 2.2%). In Bhubaneswar, ethion and endosulfan EDI was predominant. In Guwahati, EDI was utmost for fipronil, ethion and endosulfan residues with respect to TADI values for both adults and children. The Ludhiana site total acceptable daily intake (TADI) pattern also reflected the higher EDIs for fipronil, ethion and cypermethrin. In a similar way in Udaipur, the intake was highest for ethion followed by permethrin and lindane. Analyzing the available data and assuming the same dietary consumption of milk in both children and adults, it can be inferenced that children are at higher risk as compared to adults.

**Table 3:** Estimated dietary intake (EDI) (µg/day) of pesticide residues through consumption of milk from five different sites of India at lower bound (LB) and upper bound (UB) approaches.

Pesticides	ADI (ug/Kg bw/d)^a^	TADI adults (µg/d)^b^	TADI children (µg/d)^c^	Bangalore		Bhubaneswar		Guwahati		Ludhiana		Udaipur	
				LB	UB	LB	UB	LB	UB	LB	UB	LB	UB
Lindane	5	300	50	0.21	1.75	—	—	0.06	0.44	0.098	5.81	0.04	4.03
DDT	10	600	100	—	—	0.168	0.92	0.12	0.62	0.250	7.77	0.15	5.42
Endosulfan	6	360	60	0.35	2.44	0.022	0.80	0.08	0.60	—	—	—	—
Fipronil	0.2	12	2	0.33	1.71	—	—	0.02	0.37	0.78	5.91	0.08	3.77
Cyhalothrin	20	1200	200	0.03	2.14	0.04	0.82	0.06	0.58	0.64	8.29	0.04	5.40
Cypermethrin	20	1200	200	0.34	2.74	—	—	0.03	0.63	1.79	10.6	—	—
Permethrin	50	3000	500	0.09	2.60	1.152	2.07	0.13	0.75	0.304	9.48	6.76	25.9
Chlorpyrifos	10	600	100	0.48	2.37	0.135	0.83	0.05	0.53	1.62	8.52	0.38	5.88
Profenophos	30	180	300	0.27	2.3	0.038	0.79	0.02	0.53	0.80	8.25	0.07	5.34
Ethion	2	120	20	0.29	2.7	0.073	0.96	0.1	0.70	0.824	9.64	0.24	6.72

^a^Acceptable daily intake (ADI) obtained from Codex Alimentarius Commission.

^b^TADI (total acceptable daily intake) value for adults was obtained by ADI multiplied by average body weight of 60 Kg.

^c^TADI value for children was obtained by ADI multiplied by average body weight of 10 Kg.

Milk availability: Bangalore: 282 gram, Bhubeneshwar: 104 gram, Guwahati: 70 gram, Ludhiana: 1032 gram and Udaipur: 704 gram (Indiainfoline.com).

**Figure 1:**
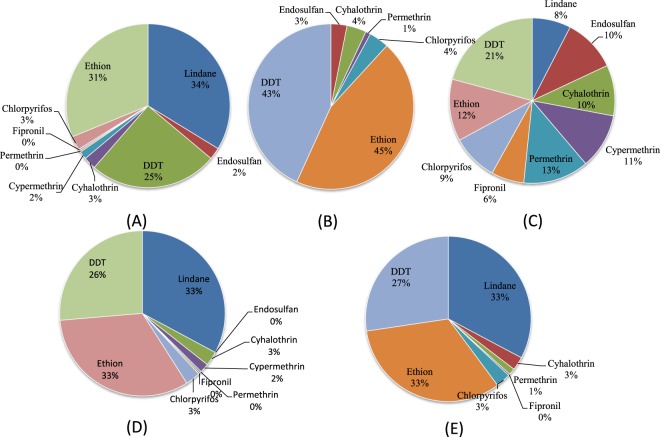
Percentage contribution of different pesticides in the Hazard index through milk consumption in Bangalore (**A**), Bhubaneswarr (**B**), Guwahati (**C**), Ludhiana (**D**) and Udaipur (**E**).

To evaluate the possible non-cancer health impact of pesticide residues through milk consumption, the hazard indices (HI) for pesticides were calculated (Table 4). It was noticed that HI values for all detectable pesticides at both lower and upper bound levels were below the target value of one in adults at all five sites in India. However, for children at the upper bound level, the HI for lindane, DDT and ethion exceeded the value of one in Ludhiana and Udaipur indicating non- cancer risks. Similar findings by Pandit and Sahu (2002) also reported that HIs fall below the target value of one at mean residue levels of HCH and DDT in India^58^. The percentage proportion of HI in total intake of residues (Fig. 1) revealed that in Bangalore the trend was lindane (34%), ethion (31%) and DDT (25%); in Bhubaneswar, ethion and DDT contributed maximum with proportion of 45 and 43 percent, respectively. In Guwahati, DDT contribution was utmost (21%), permethrin (13%) and ethion (12%); in Ludhiana, lindane, ethion and DDT proportion was 32, 31 and 27%, respectively, with similar pattern in Udaipur where the percentage proportion of lindane and ethion was 33% each followed by DDT (27%). Thus, the overall pattern indicated lindane and ethion predominance in the HI for risks to the consumer.

**Table 4:** Hazard indices for pesticide residues from milk in adults and children from five different sites of India at lower bound (LB) and upper bound (UB) approaches.

Pesticide	RfDa/µg Kg^-1^ per day ^a^	Bangalore				Bhubaneswar				Guwahati				Ludhiana				Udaipur			
		Adult		Child		Adult		Child		Adult		Child		Adult		Child		Adult		Child	
		LB	UB	LB	UB	LB	UB	LB	UB	LB	UB	LB	UB	LB	UB	LB	UB	LB	UB	LB	UB
γ-HCH	0.3	0.01	0.097	0.07	0.584	—	—	—	—	0.0031	0.024	0.02	0.147	0.005	0.323	0.03	1.937	0.002	0.224	0.06	1.34
DDT	0.5	0.005	0.073	0.03	0.041	0.005	0.032	0.03	0.19	0.004	0.04	0.02	0.24	0.008	0.271	0.048	1.627	0.005	0.187	0.13	1.120
Endosulfan	6.0	0.001	0.007	0.006	0.041	0.000	0.002	0.004	0.013	0.000	0.002	0.001	0.010	0.000	0.022	0.000	0.132	—		—	
Cyhalothrin	5.0	0.000	0.007	0.001	0.043	0.000	0.003	0.008	0.016	0.000	0.002	0.001	0.012	0.002	0.028	0.01	0.166	0.000	0.018	0.003	0.11
Cypermethrin	10.0	0.001	0.005	0.003	0.027	—		—		0.000	0.001	0.000	0.006	0.003	0.011	0.02	0.106	—		—	
Permethrin	50.0	0.000	0.001	0.0002	0.005	0.000	0.000	0.02	0.004	0.000	0.0002	0.000	0.002	0.000	0.003	0.001	0.019	0.002	0.009	0.06	0.052
Chlorpyrifos	3.0	0.002	0.008	0.010	0.047	0.000	0.003	0.003	0.017	0.000	0.0017	0.001	0.011	0.005	0.028	0.031	0.170	0.001	0.02	0.03	0.012
Ethion	0.5	0.01	0.09	0.06	0.540	0.002	0.032	0.014	0.20	0.003	0.023	0.020	0.140	0.027	0.32	0.160	1.93	0.008	0.224	0.049	1.345
Fipronil	30.0	0.000	0.001	0.001	0.006	—		—	—	0.000	0.0002	0.000	0.001	0.000	0.003	0.003	0.020	0.0000	0.002	0.001	0.013

^a^RfD values (Guidance values) obtained from USEPA.

*Estimated average daily dietary intake in ng kg^−1^ bw based on mean residue levels.

Organochlorine pesticides detected in milk and milk products have been recognized as probable human carcinogens by USEPA and IARC (International Agency for Research on Cancer). The USEPA has recommended a range of cancer risk of 01 in 10,000 to 1.0 million as acceptable, with 01 in 100,000 as typically suggested value. Comparing the cancer benchmark concentrations (BMC) calculated in present study with those derived by USEPA (Table 5), the cancer risk at lower and upper bound levels for the Indian adults were found to be with in the recommended range of reference values. However, for children though at lower bound the levels were within USEPA limits, but at upper bound levels the cancer risk (per 10,000) due to lindane was 1.3 in Bangalore, 4.4 in Ludhiana and 3.1 times in Udaipur. In a similar way, risk from DDT in children (per 10,000) was 6.4, 2.7, 3.6, 24.0 and 16.0, respectively in Bangalore, Bhubaneswar, Guwahati, Ludhiana and Udaipur. In one of the studies conducted in Punjab (1999-2001), the dietary intake of lindane was estimated 1.8 and 3.7 times higher than the ADI values based on mean and t maximum detected levels, respectively^29^. Though the dietary intake of lindane has declined now, but the concomitant intake of other pesticides (endosulfan, chlorpyrifos, cypermethrin, fipronil) may counterbalance any gains and warrants serious consideration. Further, ingestion of a mixture of pesticide residues through diet may result in more severe hazards because of the synergistic effects of the pesticides.

**Table 5:** Estimated cancer risk associated with exposure to pesticides from milk in per million populations of adults and children from five different sites of India at lower bound (LB) and upper bound (UB) approaches.

Pesticide	CSF^b^	Bangalore				Bhubaneswar				Guwahati				Ludhiana				Udaipur			
		Adult		Child		Adult		Child		Adult		Child		Adult		Child		Adult		Child	
		LB	UB	LB	UB	LB	UB	LB	UB	LB	UB	LB	UB	LB	UB	LB	UB	LB	UB	LB	UB
γ-HCH	**1300**	2.7	22.4	16.0	130	—	—	—		0.71	5.64	4.2	33.8	1.2	74	7.3	440	0.52	51	13	310
DDT	340	7.3	110	44	640	8.2	45	49	270	5.8	59	50	360	25	400	150	2400	7.6	270	190	1600

^b^Cancer SlopeFactor (ng g^-1^ per d)^-1^ as per USEPA, LB-lower bound, UB-Upper bound

In a total diet study in France, about 90% of tested pesticide’s exposure risk levels were found to be below ADI, while chronic health risks were observed for dieldrin, endrin and heptachlor intakes^59^. In Korea, the cancer risk assessment was reported in high fish intake consumers for heptachlor, dieldrin and hexachlorobenzene at upper value using probabilistic approach^60^. Darko and Akota (2008) have also reported the health risks because of chlorpyrifos, and omethioate presence in tomatoes and chlorpyrifos, dichlorvos, monocrotophos and omethioate occurrence in eggplants^61^. In general, the toxicological risk assessments are conducted on basis of a single pesticide contaminant, however potential effects due to the mixture of pesticides were not considered, further certain pesticides seen in milk do not have guidance values therefore these have not been evaluated for non-cancer and carcinogenic risk thus rendering the risk assessment of these contaminants uncertain^58^.

## Conclusions

To conclude the levels of organochlorines, organophosphates and synthetic pyrethroids were measured in the five different sites in India and cancer risks for consumers was assessed using lower bound and upper bound approaches. Results reflected the presence of HCH, DDT and endosulfan residues in some of the bovine milk samples even after the bans or restrictions on their usage. Approximately 8% milk samples were found to possess pesticide residues and violated the MRLs values. The shift of milk contamination from persistent OCPs to non- persistent OPs and SPs residues at all sites is indicating the poor agricultural and animal husbandry practices. All sites had a similar pattern of pesticides contamination in milk, except Udaipur where permethrin residues were highly dominating. Though, the current estimated dietary intakes of pesticides through bovine milk are lower than the recommended ADIs, but children were observed to be comparatively more exposed to pesticides than adults when body weight is taken into account. The exposure assessment (hazard index) in terms of cancer and non-cancer risks for pesticides lindane, DDT and ethion exceeded the recommended levels in children at upper bound levels in Ludhiana and Udaipur sites. Similarly, cancer risk was also seen high for children in all five sites at upper bound levels. On the basis of the above findings, we recommend the need for a detailed risk assessment of public health impact and regular monitoring of pesticides in milk and their products.

## Methods

### Study setting

**Figure 2:**
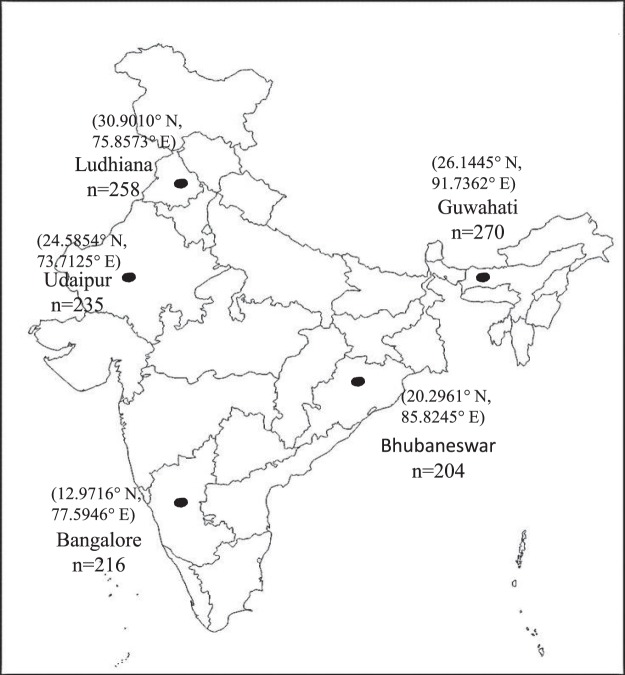
Map showing the sampling locations of peri-urban bovine milk in India.

The cross-sectional study was conducted in peri-urban fringes of five cities of India, between June 2015 and January 2016 (Fig. 2). The five sites were: Bengaluru, capital city of the state of Karnataka, located in southern part of India (12.9716°N, 77.5946°E) with population of over 10 million; Ludhiana, a large industrial city of the state of Punjab, located in northern part of India (30.9010°N, 75.8573°E) with population near to 1.5 million; Guwahati, a city of the state of Assam, in north-eastern part of India (26.1445°N, 91.7362°E) with population near to 1.0 million; Udaipur, a city in the state of Rajasthan, located in western part of India (24.5854°N, 73.7125°E) with population of 0.75 million and Bhubaneswar, capital city of Odisha, formerly Orissa, located in south-eastern part of India (20.2961°N, 85.8245°E) with population of 0.83 million^25^. We defined *peri-urban area* as a five-kilometer fringe outside of the municipal corporation boundaries of the selected site in all directions.

### Sampling

An accredited and experienced agency working in the field of GIS (geographical information system) was hired to obtain the list of villages in the peri-urban areas, as defined above. We obtained geo-coded maps of 5-kilometer peri- urban fringes from the municipality boundaries of each selected site. The maps provided detailed information of the villages and their administrative boundaries. Systematic random sampling approach was used to select sample villages at respective site. In total, 170 villages were identified in 5 sites.

A detailed census for smallholder dairy farms was conducted in all 170 villages. A list of households with smallholder dairy farms was prepared for each village. In each village, three dairy farms were selected through random sampling using the random number generator module of OpenEpi online version. This module generates a specified number of ‘random’ integers within a given range. In case of refusal or unavailability of the dairy farmer, the next farm was selected from the random number list. Once the dairy farm was selected, all the lactating cattle were given a unique identity number. A maximum of three lactating cattle were randomly selected from each dairy farm.

Milk sample was collected aseptically from each randomly selected animal using a Standard Operating Procedure (SOP). Teat disinfection/sterilization was assured prior to collection of samples. Milk sample integrity was maintained throughout by maintaining cold chain. Since, the samples were collected during normal milking of lactating animals (without any invasion) therefore, no approval from the local ethics committee was necessary.

In the field, after placing the unique identity number on the milk sample vials, each sample was zip-locked and placed in vaccine carrier boxes with ice packs at 4–8 °C. The box was then transported to the veterinary department at the respective site and samples were archived in deep freezer at −20 °C. An accredited professional agency was hired to transport the archived samples from the study sites to the central laboratory at School of Public Health and Zoonoses, Guru Angad Dev Veterinary and Animal Sciences University (GADVASU) for laboratory testing. All samples from 5 sites were transported at −20 °C and after receipt at GADVASU, were archived at −20 °C at before final processing for laboratory testing.

### Extraction of pesticide residues

In this study, milk samples were screened for 42 pesticides (parent pesticides as well as their transformation products and including metabolites). Out of these targeted pesticides, 12 are identified as priority pesticides in terms of their monitoring in food commodities. These priority pesticides are described as substances for which theoretical maximum daily intake exceeded 80% of the acceptable daily intake (ADI) for children and/or adults^62^ (chlorpyrifos, dichlorovos, dieldrin, dimethoate, endrin, ethion, heptachlor, malathion, monocrotophos, phorate, quinalphos, fenamiphos), and listed by the Stockholm Convention on persistent organic pollutants (POPs) viz. DDT, HCH, endosulfan, chlordane, mirex, dieldrin, endrin and heptachlor) and commonly used pesticides in India (n = 14, butachlor, cyhalothrin, cyfluthrin, cypermetrin, deltamethrin, fipronil, fenamiphos, fenitrothion, fenvalerate, parathion, profenophos, phosalone, permethrin, triazophos).

Pesticide residues from milk were extracted using the method developed by Battu *et al*., (2004) with slight modifications^29^. In brief, 5 ml milk was mixed with 20 grams of each activated silica gel and anhydrous sodium sulfate. The mixture was then packed into a glass column already containing dichloromethane (40 ml). After 90 minutes, the solvent in column was collected followed by elution with mixture of dichloromethane-acetone (1:2 v/v) measuring 150 ml. The extracted samples were then evaporated using rotary evaporator at 40 °C and the final reconstitution was made in 3 ml of HPLC grade n-hexane-acetone (1:1) mixture. Gas chromatograph (GC) with electron capture and flame thermionic detectors was used for the identification and quantification of pesticide residues^12^. Calibration curves for all pesticides standards were made for the concentration against area of the standard peak and the correlation coefficients (r^2^) were found to near 0.99. The analytical technical grade standards of pesticides with 93–99% purity were purchased from Sigma-Aldrich (USA).

### Method validation and quality control

The method validation comprising extraction and quantification of pesticide residues from milk was done as per the European Commission guidance document SANTE 11945/201526. Linearity of the method was evaluated by plotting a five-point linear curve (5–100 µg/kg for OCPs and SPs, 10–200 µg/kg for OPs) using three replicates of each concentration. The limit of detection (LOD) was calculated from calibration curve as: LOD = 3.3 × σ/n, N = slope of the calibration curve and σ = residual standard deviation. Since the method of extraction of pesticides involves a lot of variables which needs to controlled, an internal standard corrected calibration curve was prepared and aldrin, which has been banned in India was used as internal standard. A fixed concentration of aldrin above its LOD (i.e. 3 ng/ml) was added in each sample before extraction. The current used method was evaluated by measuring the accuracy in form of recovery percentage and precision by calculating % relative standard deviation. Recovery experiments (25, 50 and 100 µg/kg for OCPs and SPs and 25, 50, 100 and 200 µg/kg for OPs) were carried out by spiking blank milk sample with working standard solutions of pesticides. The matrix matched standards in the blank matrix sample spiked before extraction and just before analysis) were used to calculate recoveries of the target analyte. The relative standard deviation (RSD) was estimated to characterize the precision (repeatability) of the method at different fortification levels. The recovery rates of the targeted compounds were varied from 78–106% which falls within the recommended range of 70–120% (SANCO, 2009). LODs were ranged from 3.0 (aldrin, as internal standard) to ng/g to 18.6 ng/g (monocrotophos). The RSD values were found to be <15%. Selectivity of the method was assessed by analysis of blank milk sample matrix (n = 10) and reagent blank for determining any interference from the endogenous compounds nearby the retention time of the target analyte.

### Confirmation of results by GC-MS

Gas chromatography-mass spectrometry (GCMS) (Shimadzu GCMS QP2010 plus) was used for the confirmation of residues detected by GC. The GCMS was operated by setting initial column temperature at 80 °C which was finally raised to 280 °C within given time frame of the sample run. Electron ionization (EI) mode was used as ion source and the interface, manifold and ion source temperatures were set at 290 °C, 50 °C and 200 °C, respectively. The emission current of the ionization filament was fixed at 80 μA generating electrons with energy of 70 eV. Helium with purity of 99.99% was used as carrier gas at a flow rate of 0.94 ml minute^–1^. The selective ion monitoring (SIM) mode was designed for targeted pesticides including their retention time windows along with base peak ions.

### Dietary intake and risk assessment

Human exposure to pesticides from milk consumption was calculated by using concentration of pesticides in milk and the availability of milk per capita. Residue-containing samples usually do not exhibit normal distribution, and many samples contain residue contents below the limit of detection (left-censored data). Therefore, left-censored data results were treated according to the World health organization (WHO) recommendations such as substitution method that is frequently used^63^. For each target pesticide having censoring rate >60%, we assumed scenarios for left-censored results viz. the low/lower-bound (LB) and the high/upper-bound (UB) scenario.

For LB scenario, undetected results for particular pesticides were set to zero and for the UB scenario, undetected results were set to the value of LOD. It is generally expected that the LB usually underestimates the contamination and exposure levels while UB scenario overestimates these^63^.

Firstly, the dietary pesticide exposure was assessed by calculating the daily intakes of the pesticides determined in samples following the recommended guidelines for predicting dietary intake of pesticide residues of the Food and Agricultural Organization/World Health Organization^64^. The estimated daily intake (EDI) of pesticide residues was calculated using the following equation:

$${\rm{EDI}}={{\rm{C}}}_{{\rm{milk}}}\times {\rm{I}}$$

where “Cmilk” is the level of pesticide residues detected in milk and “I” is the availability of milk to an individual in grams per day at respective site. EDIs were reported in µg of pesticide/kg body weight (bw)/day. The dietary intakes of pesticides were then compared with the acceptable daily intake (ADI) recommended by FAO/WHO (2009)^65^.

In the estimation of risk assessment to consumer, Hazard Index (HI) method was used which is the sum of all Hazard Quotients (HQs) of each pesticide. The HQ was calculated from the total dietary exposure to particular pesticide (or estimated daily intake) divided by the reference dose (RfD) for the respective pesticide^66^.

$$Hazard\,Quotient=\frac{Exposure}{RfD}$$

If the HI exceeds value of one, the cumulative exposure will exceeded the maximum acceptable level and therefore there is risk of adverse health effects^67^.

For assessment of lifetime cancer risk, the average daily exposure was divided by the cancer slope factor (CSF) derived from USEPA Integrated Risk Information System for lindane and DDT where risk is the probability of lifetime cancer. This approach allowed the estimation of pesticide-specific risks for a reasonable maximum exposure using the cancer risk of 1/1,000,000 for a lifetime^68,69^. In this study, average daily doses were calculated by assuming 100% absorption of pesticides as RfDs and cancer potencies usually are not considered for bioavailability.
